# Highly oxidising fluids generated during serpentinite breakdown in subduction zones

**DOI:** 10.1038/s41598-017-09626-y

**Published:** 2017-09-04

**Authors:** B. Debret, D. A. Sverjensky

**Affiliations:** 1 Department of Earth Sciences, University of Cambridge, Downing Street, Cambridge, CB2 3EQ UK; 2 Laboratoire G-Time, DGES, Université Libre de Bruxelles, ULB, CP 160/02, 1050 Brussels, Belgium; 3 Department of Earth and Planetary Sciences, Johns Hopkins University, Baltimore, Maryland 21218 USA

## Abstract

Subduction zones facilitate chemical exchanges between Earth’s deep interior and volcanism that affects habitability of the surface environment. Lavas erupted at subduction zones are oxidized and release volatile species. These features may reflect a modification of the oxidation state of the sub-arc mantle by hydrous, oxidizing sulfate and/or carbonate-bearing fluids derived from subducting slabs. But the reason that the fluids are oxidizing has been unclear. Here we use theoretical chemical mass transfer calculations to predict the redox state of fluids generated during serpentinite dehydration. Specifically, the breakdown of antigorite to olivine, enstatite, and chlorite generates fluids with high oxygen fugacities, close to the hematite-magnetite buffer, that can contain significant amounts of sulfate. The migration of these fluids from the slab to the mantle wedge could therefore provide the oxidized source for the genesis of primary arc magmas that release gases to the atmosphere during volcanism. Our results also show that the evolution of oxygen fugacity in serpentinite during subduction is sensitive to the amount of sulfides and potentially metal alloys in bulk rock, possibly producing redox heterogeneities in subducting slabs.

## Introduction

During subduction, the increase of pressure and temperature conditions in the subducting plate results in hydrous mineral breakdown and the release of volatile-rich fluids. The oxidized or reduced nature of the released fluids is controlled by mineral local equilibrium changes and can be monitored through the oxygen fugacity (*f*O_2_). Although large variabilities of *f*O_2_ were shown to occur in subduction zones, especially in melange zones constituting the plate/mantle interface^1^, previous petrological and geochemical studies have shown that the redox state of the subducted mafic crust does not change during prograde metamorphism and that its redox budget remains relatively constant^2^. Therefore, other sources of fluids must be considered to explain the oxidized nature of arc magmas^3–5^.

Serpentinized mantle peridotites can constitute a major source of water at depth in subduction zones^6^. Those rocks comprise a significant part of subducting oceanic lithosphere hydrated near the seafloor at slow or ultra-slow spreading ridges^7, 8^ or slab-bending related faults^9^, and are also present as part of the slab-wedge interface or mantle wedge that is percolated by aqueous fluids emanating from the slab during subduction^10, 11^. Serpentinites are thus ubiquitous in subduction zones. Furthermore, several studies have suggested that the breakdown of these rocks is the most likely source of fluid-mobile elements (e.g. B), volatiles and halogens enriched in arc magmas (e.g. ^12–17^). The dehydration of serpentinized mantle results in a net decrease in bulk rock volatile concentrations^6, 16, 17^ and Fe^3+^/∑e^18, 19^. In addition, observations of hematite-magnetite assemblages in dehydrated serpentinites^20, 21^ suggest a high *f*O_2_, from one to five log units above the Quartz-Fayalite-Magnetite (QFM) oxygen buffer, during antigorite breakdown in subduction zones which could be compatible with the release of oxidized S and C in slab-derived fluids. If correct, this model would have major implications for the cycling of volatiles between the Earth’s surface and interior, volcanic degassing (e.g. SO_X_, CO_X_ species emitted from volcanoes), and the occurrence of metal sulfide ore deposits^22, 23^. Although several studies have indicated some evidence of variations of *f*O_2_ in antigorite-bearing serpentinites that can be related to the extent of initial serpentinization^24, 25^, there is at this time no consensus on the evolution of *f*O_2_ or the redox state of serpentinite-derived fluids during antigorite breakdown in subduction zones.

Recent advances in theoretical and experimental aqueous geochemistry resulting in the Deep Earth Water (DEW) model now enable the calculation of equilibrium constants involving minerals and aqueous ions, metal complexes, and organics to 6.0 GPa and 1,200 °C^26–33^. In the present study, we model the dehydration of antigorite-bearing serpentinite to investigate its potential to liberate highly oxidizing fluids in subduction zones. The model uses a conceptual scenario in which an initial fluid chemistry was set by reaction of water with an antigorite-bearing serpentinite at 630 °C before undergoing an increase of temperature to 660 °C at constant pressure. The initial model assemblage is composed of antigorite, olivine (XMg [Mg/(Fe + Mg)] = 0.885), clinochlore, magnetite, and tremolite in agreement with field observations from Padron-Navarta *et al*.^34^. This assemblage sets the initial *f*O_2_ which ranges from near QFM at 500 °C and 2.0 GPa to QFM + 4.2 at 650 °C and 2.0 GPa (Supp. Info Fig. S1). At the lowest temperature in this range the *f*O_2_ near QFM is in agreement with recent work on natural samples^24^. However, at 630 °C the *f*O_2_ is several units above QFM just before the breakdown of antigorite. In this study, we explore the further dramatic *f*O_2_ changes on heating through the breakdown of antigorite.

## Modelling serpentinite dehydration during subduction

**Figure 1:**
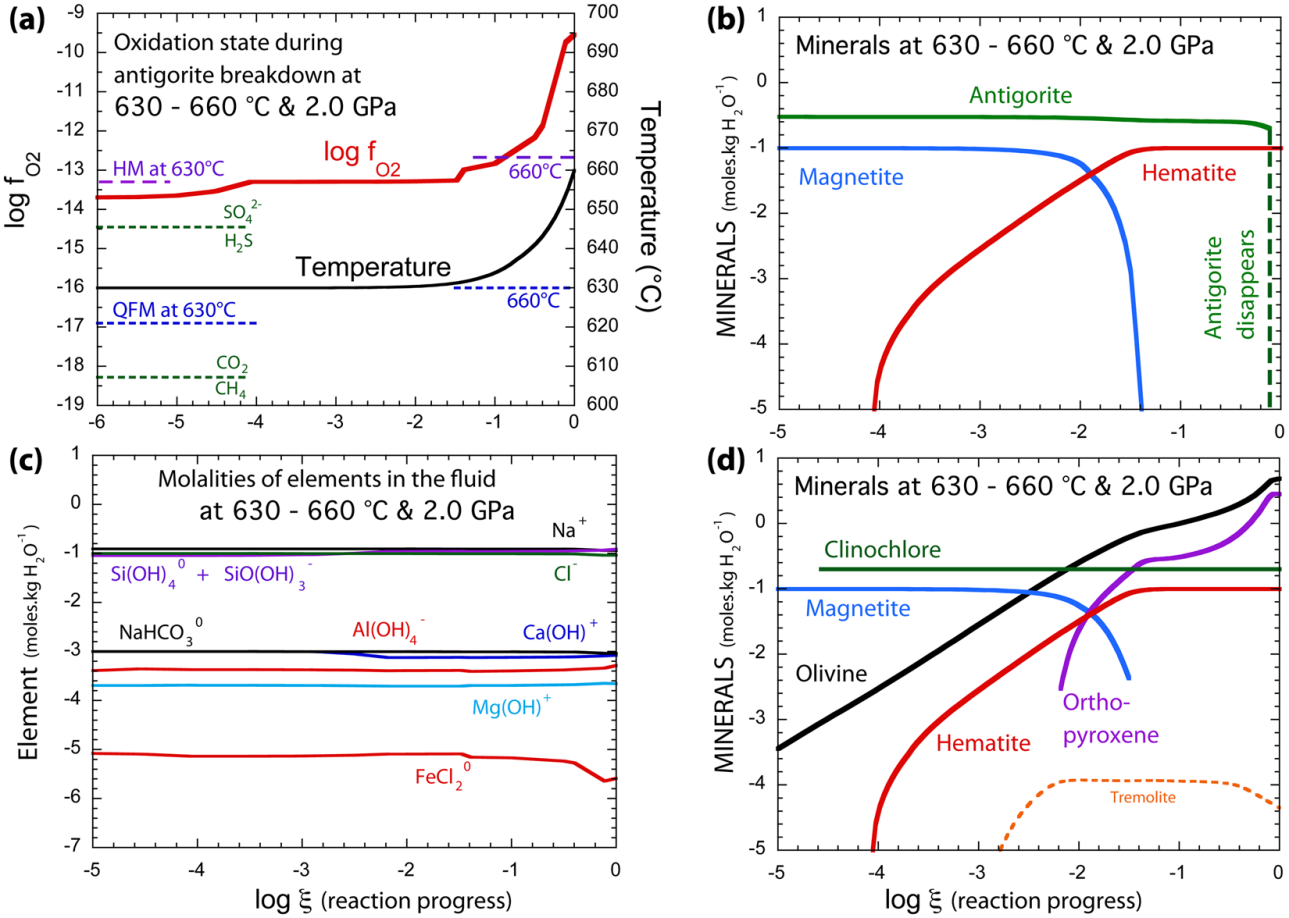
Predicted evolution of *f*O_2_, mineral reactants, aqueous phase composition, and mineral products during antigorite breakdown at 2 GPa in a sulfur-free model. (**a**) Evolution of temperature and *f*O_2_ in the system. QFM and magnetite-hematite buffers are reported in dashed lines. (**b**) Abundances of key minerals involved.Hematite appears at the beginning of the reaction and remains stable until the disappearance of antigorite and magnetite. (**c**) Concentrations of major species in the aqueous fluid. (**d**) Minerals produced during antigorite breakdown. The x axis represents the logarithm of the reaction progress variable ξ, which is equal to the number of moles of each reactant mineral destroyed during the reaction progress.

Figure 1 displays the evolution of the *f*O_2_, mineral assemblages, and fluid composition during antigorite breakdown at 2 GPa (modelling carried out at 4 GPa displays a similar evolution as presented in Supp. Info Fig. S2). In agreement with natural^21, 34^ observations, the reaction progress leads to the successive growth of olivine, clinochlore, and orthopyroxene and the progressive decrease of the amount of magnetite (Fig. 1b,d). During the first part of the reaction path (logξ < −4), the crystallization of olivine and chlorite is accompanied by a progressive increases of *f*O_2_ up to four log units above QFM oxygen buffer (Fig. 1a). At these conditions, hematite becomes in equilibrium with magnetite and buffers the *f*O_2_. The reaction progress is then accompanied with a progressive decrease of the amount of magnetite and the appearance of orthopyroxene (^+^/^−^ tremolite) in the rock (Fig. 1d). A second increase of *f*O_2_ appears later (logξ > −1.4) and corresponds to the disappearance of magnetite (Fig. 1a,b).

Few variations in pH and the composition of the released fluids (Fig. 1c) are observed during the reaction progress. The pH is alkaline and varies slightly around 5 (neutral pH is about 3.4 at these conditions; Supp. Info Fig. S3), while the aqueous species are dominated by Na^+^_(aq)_, Cl^−^_(aq)_ and Si(OH)_4(aq)_ and SiO(OH)_3_^−^_(aq)_, with minor amounts of NaHCO_3(aq)_, Al(OH)_4_^−^_(aq)_, Ca(OH)^+^_(aq)_, Mg(OH)^+^_(aq)_, and FeCl_2(aq)_. The presence of equilibrated hematite-magnetite assemblages in our models is compatible with recent observations in meta-peridotites from the Cerro Del Almirez massif^21^ or partly dehydrated serpentinites from Western Alps meta-ophiolites^20^. It therefore suggests that the dehydration of serpentinites in subduction zones can take place at high *f*O_2_, close to the hematite-magnetite buffer. At such *P*-*T*-*f*O_2_ *-*pH conditions, volatile and redox-sensitive elements, such as sulfur or carbon, are expected to be mobilized under in their oxidized form (e.g. CO_2_^0^_aq)_ or SO_4_^2−^_(aq)_) rather than reduced species (e.g. CH_4(aq)_ or HS^−^_(aq)_; Fig. 1a).

## The importance of sulfides

**Figure 2:**
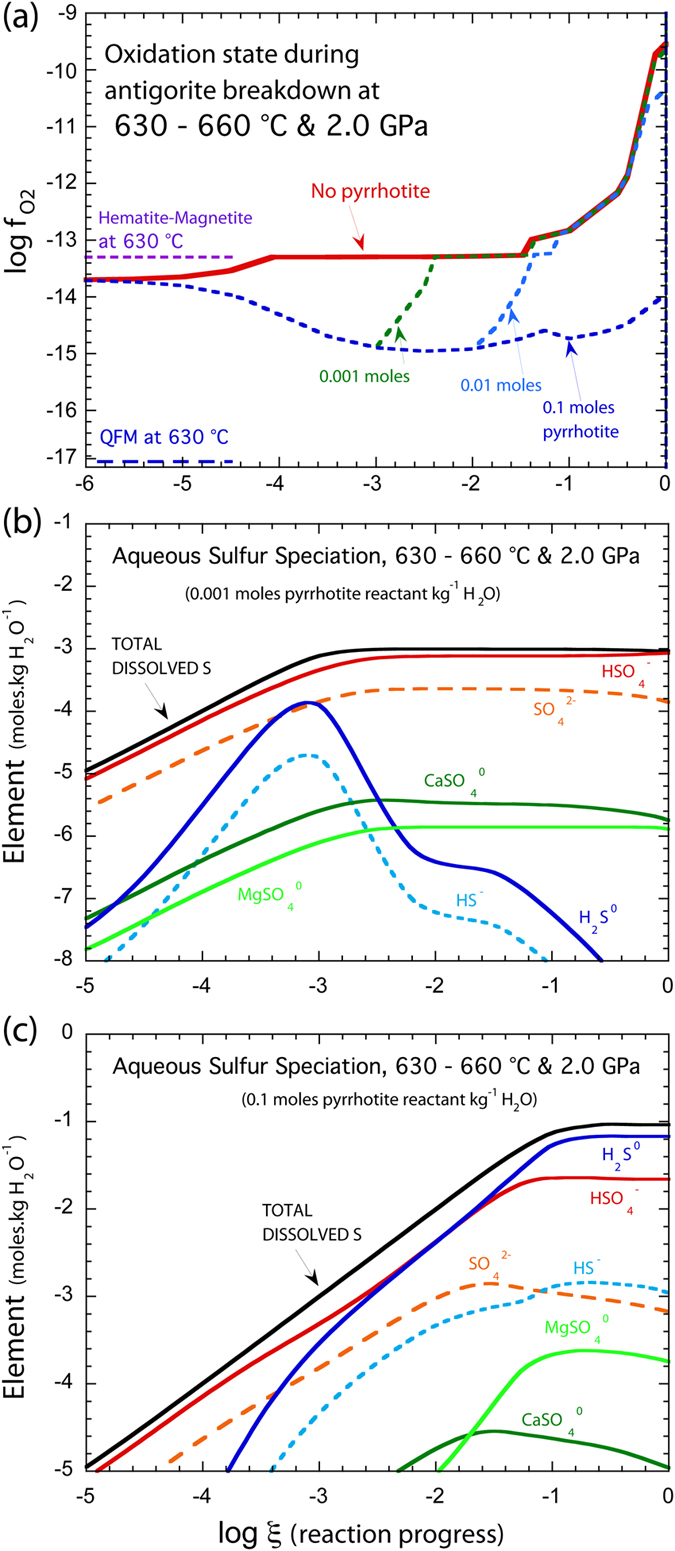
Predicted evolution of *f*O_2_ and aqueous phase composition in the presence of pyrrhotite during antigorite breakdown at 2 GPa. (**a**) Evolution of *f*O_2_ for different amounts of pyrrhotite (No pyrrhotite, 0.001, 0.01 and 0.1 moles). (**b**,**c**) Number of moles of sulfur dissolved into the fluid per kg of water with the evolution of major aqueous sulfur species. In b and c, the amount of sulfur present in the initial assemblage varies from 0.001 to 0.1 moles of pyrrhotite.

Although the progressive increase in *f*O_2_ during prograde metamorphism is suggestive of oxidized fluids release during antigorite breakdown, previous studies have shown that the presence of Ni-Fe alloys and/or sulfides can buffer the *f*O_2_ down to four or five log units below the QFM buffer in serpentinites^35, 36^. In addition, several studies have shown that sulfides can persist^17^ or even be formed^24, 25^ during serpentinite devolatilization. In order to test the impact of sulfur-bearing phases during the progressive devolatilization of serpentinites in subduction zones, we ran a suite of models containing various amounts of pyrrhotite (0.001, 0.01 and 0.1 moles), which is commonly observed in abyssal and orogenic serpentinites^17^. The most pronounced difference between sulfide-free and sulfide-bearing models is the evolution of *f*O_2_ during the reaction progress (Fig. 2a). The presence of a relatively small amount of pyrrhotite (0.001 to 0.01 moles of pyrrhotite) is accompanied with a decrease of *f*O_2_ from three to two log units above QFM buffer (logξ < −3 or −2, Fig. 2a). This stage is associated with the total destruction of the pyrrhotite and an increase of sulfur concentration in the serpentinite-derived fluids. Interestingly, even if hematite is absent from the system at this stage, sulfate bearing species (HSO_4_^−^_(aq)_, SO_4_^2−^_(aq)_, CaSO_4 (aq)_, MgSO_4 (aq)_) are dominating the fluid composition relative to sulfide bearing species (H_2_S and HS^−^, Fig. 2b). This suggests that even at modestly oxidizing *f*O_2_ values, sulfates are continuously released in serpentinite-derived fluids. As the reaction progresses (from logξ of −3 to −1.4), the destruction of sulfide and then magnetite is accompanied by an increase of *f*O_2_ and the appearance of hematite (Fig. 2b). In these conditions, sulfide species are at negligible concentrations in the fluid phase which is mainly composed of HSO_4_^−^_(aq)_, SO_4_^2−^_(aq)_, CaSO_4 (aq)_ and MgSO_4 (aq)_. For high sulfide concentrations (0.1 mole of pyrrhotite), aqueous sulphide species remain at significant concentrations in the fluid during the whole reaction progress and become more abundant than sulphate species above logξ of about −1.5 (Fig. 2c). These species buffer the *f*O_2_ at three units above QFM buffer but below the Hematite-Magnetite buffer, therefore preventing hematite crystallization (Fig. 2a). However, it should be noted that, even if the presence of these high amounts of pyrrhotite buffer the *f*O_2_ at relatively low values, the amount of sulfur and sulfate dissolved in the fluid is significantly higher in those models (Fig. 2b and c).

## Evolution of oxygen fugacity and implications for the nature of slab derived fluids

Iron, carbon and sulfur are the main redox sensitive elements that can be transferred by fluids from the slab to the mantle wedge, having thus the potential to modify the redox state of the source region of arc magmas above subduction zones^4, 5, 37^. In a previous Fe isotope study on subducted meta-ophiolites, it was shown that Fe can be mobile in slab-derived fluids in the form of Fe(II)-Cl_2_ and/or Fe(II)-SO_X_^20^. In good agreement with these observations, thermodynamic calculations predict that during serpentinite dehydration, the magnetite modal amount significantly decreases (Fig. 1a,b) while the amount of Fe release in the fluid remains very low (Fig. 1c) and is dominated by the FeCl_2_ species. We note that the complexation of Fe(II) to sulfate is not considered in the DEW model and even though Fe(III)-complexes are included, they are completely unimportant at the relatively high temperatures and low chloride concentrations of the present study. In other words, with our present state of knowledge of aqueous Fe-complexes, there are none known that could contribute significantly to the transport of Fe(III). It should also be noted that chlorine concentrations in fluid inclusions hosted by metamorphic olivines range up to 2 wt% NaCl. Such chlorine concentrations could potentially lead to a higher mobility of Fe(II) in serpentinites-derived fluids^14, 15^.

Carbon is mainly transported via CO_2,aq_ under the conditions we investigated in subduction zones. Sources of this C could be in sediments and ophicarbonates (up to 3 wt%; e.g. refs 38 and 39). Although there are few constraints on carbon mobility during serpentinite dehydration, eclogitic serpentinites and meta-peridotites from Almirez massif display similar low concentrations (between 200 and 800 ppm) and preserve δ^13^C and C consistent with mixing at low pressure between reduced carbon and seawater derived carbonates^40^. It thus suggests that carbon is not the main oxidizing agent release by slab serpentinite dehydration at high pressure. Instead, the sulfur concentrations of subducted ultramafic rocks are highly variable (from about 50 ppm to 2000 ppm) reflecting a mobility of sulfur in serpentinite-derived fluids during subduction^40, 41^. Indeed the presence of sulfur-bearing daughter phases precipitating after fluid entrapment in dehydrated serpentinite shows a significant mobility of sulfur in serpentinite derived fluids during subduction^15^. In agreement with these studies, sulfur-bearing models reveal the total destruction of sulfur-bearing phases during the first stages of serpentinite dehydration and the presence of sulfate in serpentinite-derived fluids. The ratio of total sulfate over total sulfur in serpentinite derived fluids increases with the *f*O_2_ (Fig. 2b and c).

The *f*O_2_ evolution of serpentinites during antigorite breakdown is clearly highly influenced by pre-subduction characteristics of the rocks, specifically the initial assemblage (e.g. modal amount of sulfide, Fig. 2a). At mid-oceanic ridges, serpentinites formed in the deep part of the lithosphere or nearby metagabbroic intrusions are likely to contain a high amount of mantle sulfides^17, 42^ and other reduced species such as awaruite^25^. In contrast, serpentinites sampled at the seafloor level are formed at high water-rock ratio and can contain a large amount of sulfate^14, 42^. Such large-scale heterogeneities are likely to be preserved during subduction^43^ resulting in a variety of mineralogical and *f*O_2_ evolution pathways in ultramafic rocks during oceanic lithosphere dehydration. Indeed, the existence of subducted serpentinites equilibrated at variable *f*O_2_, from −3 to + 5^21, 25^, in different subduction settings is in good agreement with this scenario. It thus suggests that in the deep and/or less serpentinized parts of the subducted lithosphere, the presence of reduced assemblages (e.g. awaruite, sulfide) can buffer the *f*O_2_ to relatively low values, until their eventual destruction during antigorite breakdown, while toward to the top of the slab and/or nearby slab-bending related fractures the ultramafic rocks will preferentially crystallize assemblages equilibrated at high *f*O_2_. As a consequence, the *f*O_2_ of the slab is likely to be heterogeneous, reflecting different pre-subduction mineralogy. However, in highly serpentinized peridotites, regardless of the considered models, sulfur seems to be highly mobile in fluids during antigorite breakdown (Fig. 2b,c). Interestingly, although sulfide can buffer the *f*O_2_ to relatively modest elevations above QFM, sulfate-bearing species (HSO_4_^−^_(aq)_, SO_4_^2−^_(aq)_, CaSO_4 (aq)_, MgSO_4 (aq)_) always represent a significant proportion of the sulfur-bearing phases carried by the fluid. The migration of these fluids from the slab to the slab-mantle interface or mantle wedge can therefore enhance the oxidation of the mantle wedge Fe^2+^ to Fe^3+^ in response to the reduction of slab fluid SO_4_^2−^ to S^2−^. Such processes may provide an oxidized mantle source region for primary arc magmas. In this way, the origin of the oxidized signature carried by fluids from the slab may be an inevitable consequence of the breakdown of antigorite during subduction.

## Electronic supplementary material

Supplementary Figures

## References

[1] Tumiati S, Godard G, Martin S, Malaspina N, Poli S (2015). Ultra-oxidized rocks in subduction melanges? Decoupling between oxygen fugacity and oxygen availability in a Mn-rich metasomatic environment. Lithos.

[2] Li, J.-L., Gao, J., Klemd, R., John, T. & Wang, X.-S. Redox processes in subducting oceanic crust recorded by sulfide-bearing high-pressure rocks and veins (SW Tianshan, China). *Contributions to Mineralogy and Petrology* **171**, doi:10.1007/s00410-016-1284-2 (2016).

[3] Parkinson IJ, Arculus RJ (1999). The redox state of subduction zones: insights from arc-peridotites. Chemical Geology.

[4] Kelley KA, Cottrell E (2009). Water and the Oxidation State of Subduction Zone Magmas. Science.

[5] Evans KA (2012). The redox budget of subduction zones. Earth-Science Reviews.

[6] Ulmer P, Trommsdorff V (1995). Serpentine Stability to Mantle Depths and Subduction-Related Magmatism. Science.

[7] Canales JP, Collins JA, Escartin J, Detrick RS (2000). Seismic structure across the rift valley of the Mid-Atlantic ridge at 23◦20_N (MARK area): implications for crustal accretion processes at slow-spreading ridges. Journal of Geophysical Research.

[8] Andreani M, Mével C, Boullier A-M, Escartin J (2007). Dynamic control on serpentine crystallization in veins: constraints on hydration processes in oceanic peridotites. Geochemistry Geophysics Geosystems.

[9] Ranero CR, Morgan JP, McIntoch K, Reichert C (2003). Bending-related faulting and mantle serpentinization at the Middle America trench. Nature.

[10] Hattori KH, Guillot S (2007). Geochemical character of serpentinites associated with high- to ultrahigh-pressure metamorphic rocks in the Alps, Cuba, and the Himalayas: recycling of elements in subduction zones. Geochemistry Geophysics Geosystems.

[11] Reynard B (2013). Serpentine in active subduction zones. Lithos.

[12] Tenthorey E, Hermann J (2004). Composition of fluids during serpentinite breakdown in subduction zones: evidence for limited boron mobility. Geology.

[13] Kendrick MA, Scambelluri M, Honda M, Phillips D (2011). High abundances of noble gas and chlorine delivered to the mantle by serpentinite subduction. Nature Geoscience.

[14] Scambelluri M, Fiebig J, Malaspina N, Müntener O, Pettke T (2004). Serpentinite Subduction: Implications for Fluid Processes and Trace-Element Recycling. International Geology Review.

[15] Scambelluri M, Pettke T, Cannaò E (2015). Fluid-related inclusions in Alpine high-pressure peridotite reveal trace element recycling during subduction-zone dehydration of serpentinized mantle (Cima di Gagnone, Swiss Alps). Earth and Planetary Science Letters.

[16] Scambelluri M, Tonarini S (2012). Boron isotope evidence for shallow fluid transfer across subduction zones by serpentinized mantle. Geology.

[17] Alt JC (2013). The role of serpentinites in cycling of carbon and sulfur: Seafloor serpentinization and subduction metamorphism. Lithos.

[18] Debret B (2014). Evolution of Fe redox state in serpentine during subduction. Earth and Planetary Science Letters.

[19] Merkulova M, Muñoz M, Vidal O, Brunet F (2016). Role of iron content on serpentinite dehydration depth in subduction zones: Experiments and thermodynamic modeling. Lithos.

[20] Debret B (2016). Isotopic evidence for iron mobility during subduction. Geology.

[21] Debret, B. *et al*. Redox state of iron during high-pressure serpentinite dehydration. *Contributions to Mineralogy and Petrology* **169**, doi:10.1007/s00410-015-1130-y (2015).

[22] Gaillard F, Scaillet B, Pichavant M, Iacono-Marziano G (2015). The redox geodynamics linking basalts and their mantle sources through space and time. Chemical Geology.

[23] Frost DJ, McCammon CA (2008). The redox state of Earth’s mantle. Annual Review of Earth and Planetary Sciences.

[24] Evans KA, Powell R (2015). The effect of subduction on the sulphur, carbon and redox budget of lithospheric mantle. Journal of Metamorphic Geology.

[25] Evans KA, Reddy SM, Tomkins AG, Crossley RJ, Frost BR (2017). Effects of geodynamic setting on the redox state of fluids released by subducted mantle lithosphere. Lithos.

[26] Sverjensky DA, Stagno V, Huang F (2014). Important role for organic carbon in subduction-zone fluids in the deep carbon cycle. Nat Geosci.

[27] Sverjensky DA, Harrison B, Azzolini D (2014). Water in the deep Earth: The dielectric constant and the solubilities of quartz and corundum to 60 kb and 1200 degrees C. Geochim Cosmochim Ac.

[28] Pan D, Spanu L, Harrison B, Sverjensky DA, Galli G (2013). Dielectric properties of water under extreme conditions and transport of carbonates in the deep Earth. P Natl Acad Sci USA.

[29] Facq S, Daniel I, Montagnac G, Cardon H, Sverjensky DA (2014). *In situ* Raman study and thermodynamic model of aqueous carbonate speciation in equilibrium with aragonite under subduction zone conditions. Geochim Cosmochim Ac.

[30] Mikhail S, Sverjensky DA (2014). Nitrogen speciation in upper mantle fluids and the origin of Earth’s nitrogen-rich atmosphere. Nature Geoscience.

[31] Sverjensky DA, Huang F (2015). Diamond formation due to a pH drop during fluid-rock interactions. Nature Communications.

[32] Facq S, Daniel I, Montagnac G, Cardon H, Sverjensky DA (2016). Carbon speciation in saline solutions in equilibrium with aragonite at high pressure. Chemical Geology.

[33] Mikhail S, Barry P, Sverjensky DA (2017). The role of pH in the deep-Earth nitrogen cycle. Geochimica et Cosmochimica Acta.

[34] Padron-Navarta JA, Sanchez-Vizcaino VL, Garrido CJ, Gomez-Pugnaire MT (2011). Metamorphic Record of High-pressure Dehydration of Antigorite Serpentinite to Chlorite Harzburgite in a Subduction Setting (Cerro del Almirez, Nevado-Filabride Complex, Southern Spain). J Petrol.

[35] Frost BR (1985). On the Stability of Sulfides, Oxides, and Native Metals in Serpentinite. J Petrol.

[36] Klein F, Bach W (2009). Fe-Ni-Co-O-S Phase Relations in Peridotite-Seawater Interactions. J Petrol.

[37] Evans KA, Tomkins AG (2011). The relationship between subduction zone redox budget and arc magma fertility. Earth and Planetary Science Letters.

[38] Gorman PJ, Kerrick DM, Connolly JAD (2006). Modeling open system metamorphic decarbonation of subducting slabs. Geochem Geophys Geosyst.

[39] Kelemen, P. B. & Manning, C. E. Reevaluating carbon fluxes in subduction zones, what goes down, mostly comes up. *P Natl Acad Sci USA* **113**, doi:10.1073/pnas.1507889112 (2015). 10.1073/pnas.1507889112 PMC4522802 26048906

[40] Alt JC (2012). Recycling of water, carbon, and sulfur during subduction of serpentinites: A stable isotope study of Cerro del Almirez, Spain. Earth and Planetary Science Letters.

[41] Pons, M. L., Debret, B., Bouilhol, P., Delacour, A. & Williams, H. Zinc isotope evidence for sulfate-rich fluid transfer across subduction zones. *Nature Communication* **13794**, doi:10.1038/ncomms13794 (2016). 10.1038/ncomms13794 PMC5171646 27982033

[42] Debret B (2017). Assessing sulfur redox state and distribution in abyssal serpentinites using X-ray absorption spectroscopy. Earth and Planetary Science Letters.

[43] Debret B, Nicollet C, Andreani M, Schwartz S, Godard M (2013). Three steps of serpentinization in an eclogitized oceanic serpentinization front (Lanzo Massif - Western Alps). Journal of Metamorphic Geology.

