# Supplementary material for: Highly oxidising fluids generated during serpentinite breakdown in subduction zones
Source: Sci Rep. 2017 Sep 4;7:10351. doi: 10.1038/s41598-017-09626-y (PMC5583334; doi:10.1038/s41598-017-09626-y)
Supplement: Supplementary file 1 — Supplementary Figures [file 41598_2017_9626_MOESM1_ESM.pdf]

Highly oxidising fluids generated during serpentinite breakdown in subduction zones

Supplementary Information

B. Debret[1,2*] & D. A. Sverjensky[3]

1: Department of Earth Sciences, University of Cambridge, Downing Street, Cambridge CB2 3EQ, UK

2: Laboratoire G-Time, DGES, Université Libre de Bruxelles, ULB, CP 160/02, 1050 Brussels, Belgium

3: Department of Earth and Planetary Sciences, Johns Hopkins University, Baltimore, Maryland 21218, USA

**Correspondence to ba.debret@gmail.com*

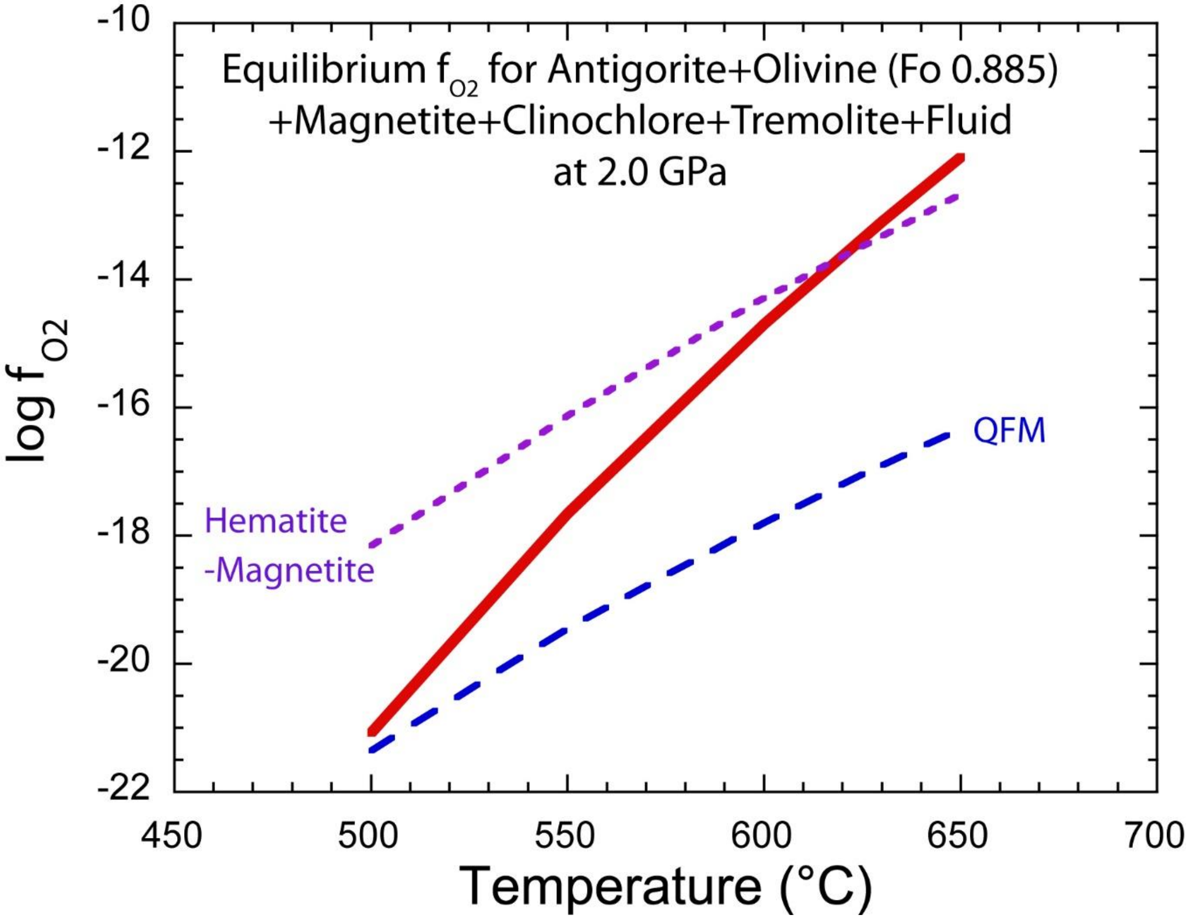

Figure S1: Predicted f_{O2} evolution of the initial antigorite, olivine (Fo 0.885), magnetite, clinochlore, tremolite, and fluid assemblage (red line) with temperature. At 630°C, the composition of the initial assemblage is equilibrated at high f_{O2} relative to seafloor serpentinites (e.g. Evans, 2008). Indeed, during the first stages of subduction, the transition from lizardite to antigorite (around 300 to 400°C) is accompanied by a reduction of Fe and the recrystallization of seafloor assemblages. This redox reaction might be coupled with the oxidation of reduced oceanic phases such as sulfides or awaruite, and the formation of oxidized fluids (e.g. SO_X , H_2O, CO_X) resulting in an increase of f_{O2} in the slab (Debret et al., 2014; Evans and Powell, 2015; Pons et al., 2016).

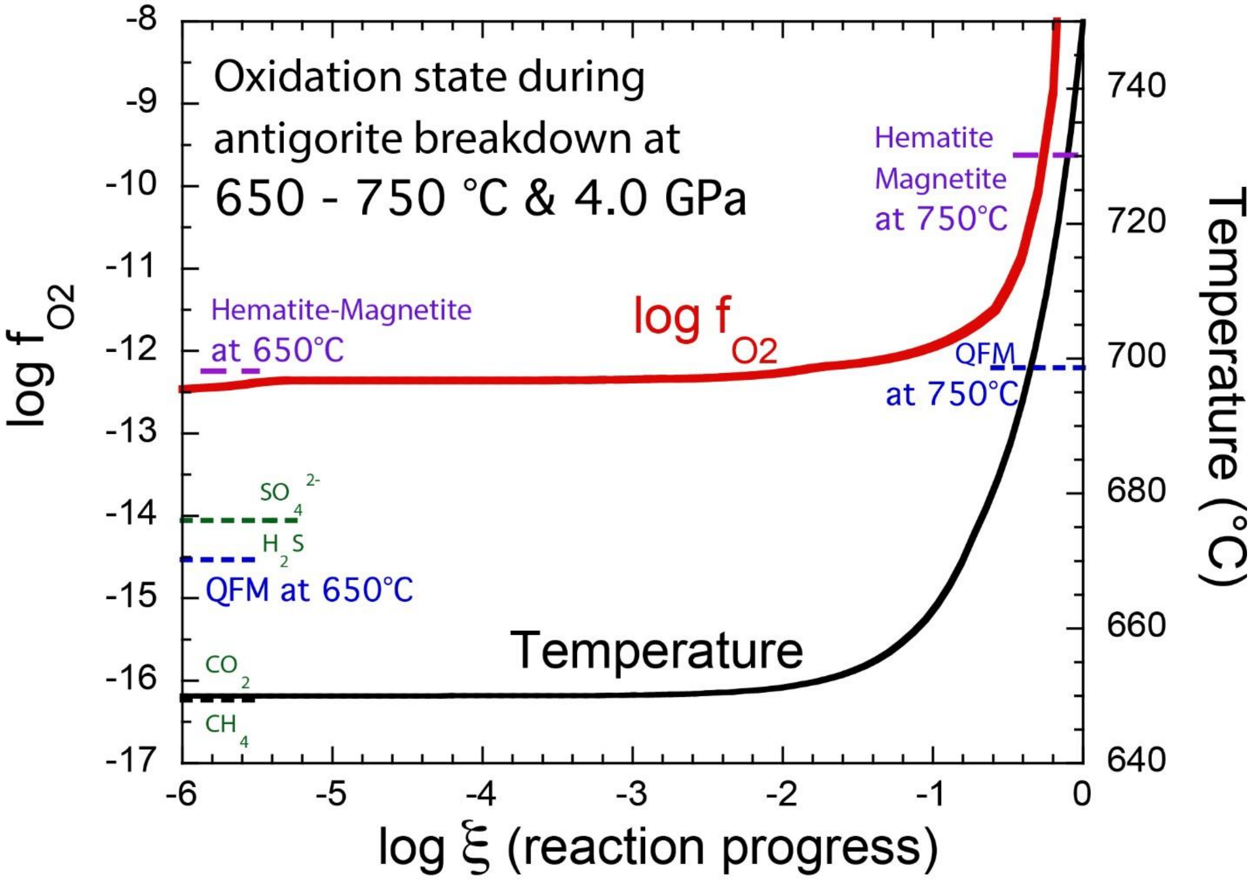

Figure S2: Predicted evolution of f_{O2} during antigorite breakdown at 4 GPa in sulfur-free models. No significant modification of f_{O2} evolution has been observed with pressure increase, suggesting that the behaviour of the system is mainly temperature dependent.

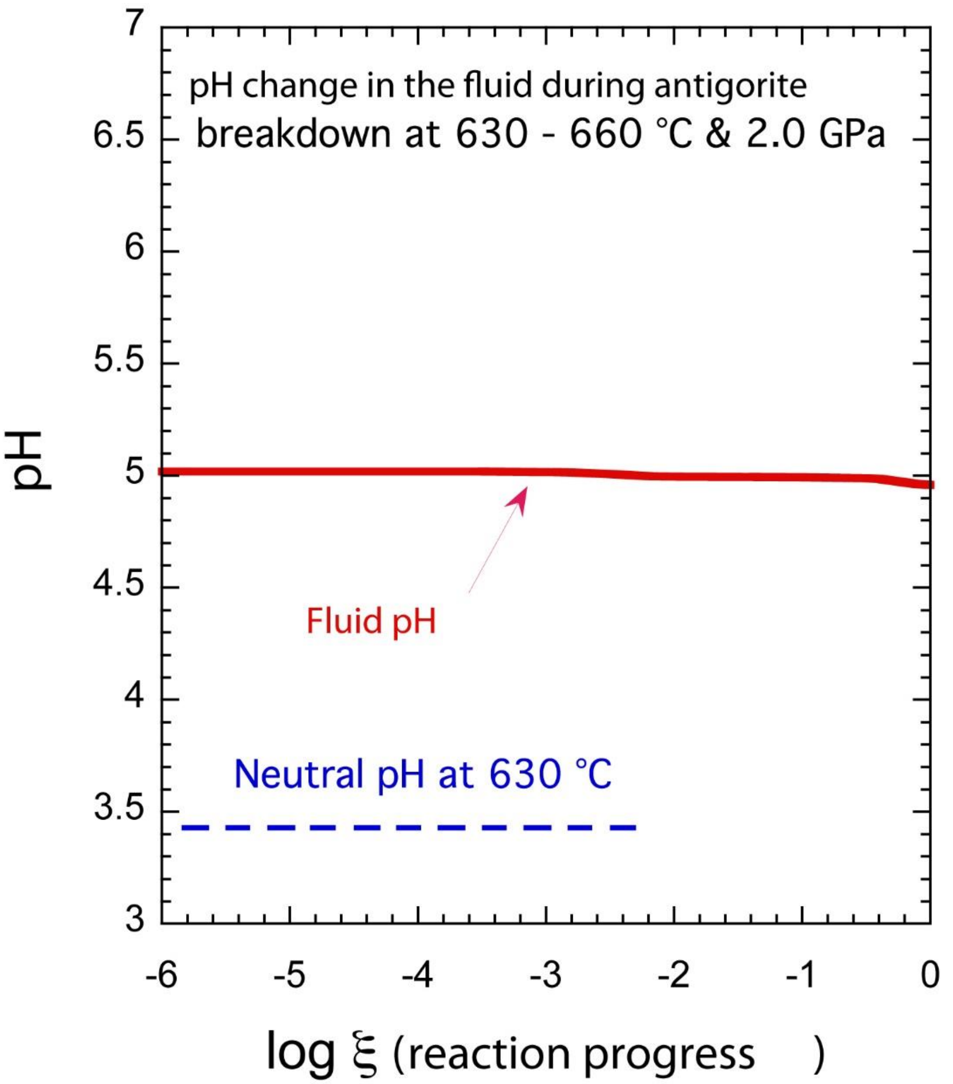

Figure S3: Predicted pH evolution during antigorite breakdown at 2 GPa in sulfur-free models. The pH remains relatively constant and is alkaline (neutral pH is about 3.4 at these conditions).
